# Exploring the impact of evaluation on learning and health innovation sustainability: protocol for a realist synthesis

**DOI:** 10.1186/s13643-023-02348-5

**Published:** 2023-10-06

**Authors:** Marissa Bird, Élizabeth Côté-Boileau, Walter P. Wodchis, Lianne Jeffs, Maura MacPhee, James Shaw, Tujuanna Austin, Frances Bruno, Megan Bhalla, Carolyn Steele Gray

**Affiliations:** 1 Institute for Better Health, 100 Queensway West–Clinical, Administrative Building, 6Th Floor, Mississauga, ON L5B 1B8 Canada; 2 Institute of Health Policy Management and Evaluation (IHPME), Dalla Lana School of Public Health, University of Toronto, Suite 425–155 College Street, Toronto, ON M5T 3M6 Canada; 3 Department of Health Management, Evaluation and Policy, School of Public Health, University of Montreal, 7101 Av du Parc, Montréal, QC H3N 1X9 Canada; 4 Science of Care Institute, Sinai Health, 1 Bridgepoint Drive, Toronto, ON M4M 2B5 Canada; 5 Nursing-Applied Sciences, University of British Columbia, 239-2211 Wesbrook Mall, Vancouver, BC V6T2B5 Canada; 6 Department of Physical Therapy, Temerty Faculty of Medicine, University of Toronto, 500 University Ave, Toronto, ON M5G 1V7 Canada; 7 OPTI-Hex Lab, Leslie Dan Faculty of Pharmacy, University of Toronto, 144 College St, Toronto, ON M5S 3M2 Canada

**Keywords:** Realist synthesis, Evaluation, Learning, Learning health systems, Innovation, Sustainability

## Abstract

**Background:**

Within the Learning Health System (LHS) model, learning routines, including evaluation, allow for continuous incremental change to take place. Within these learning routines, evaluation assists in problem identification, data collection, and data transformation into contextualized information, which is then re-applied to the LHS environment. Evaluation that catalyzes learning and improvement may also contribute to health innovation sustainability. However, there is little consensus as to why certain evaluations seem to support learning and sustainability, while others impede it. This realist synthesis seeks to understand the contextual factors and underlying mechanisms or drivers that best support health systems learning and sustainable innovation.

**Methods:**

This synthesis will be guided by Pawson and colleagues’ 2005 and Emmel and colleagues’ 2018 guidelines for conducting realist syntheses. The review process will encompass five steps: (1) scoping the review, (2) building theories, (3) identifying the evidence, (4) evidence selection and appraisal, and (5) data extraction and synthesis. An Expert Committee comprised of leaders in evaluation, innovation, sustainability, and realist methodology will guide this synthesis. Review findings will be reported using the RAMESES guidelines.

**Discussion:**

The use of a realist review will allow for exploration and theorizing about the contextual factors and underlying mechanisms that make evaluations ‘work’ (or ‘not work’) to support learning and sustainability. Depending on results, we will attempt to synthesize findings into a series of recommendations for evaluations with the intention to support health systems learning and sustainability. Finalized results will be presented at national and international conferences, as well as disseminated via a peer-reviewed publication.

**Systematic review registration:**

This realist synthesis protocol has been registered with PROSPERO (https://www.crd.york.ac.uk/prospero/ ID 382690).

**Supplementary Information:**

The online version contains supplementary material available at 10.1186/s13643-023-02348-5.

## Background

Learning Health Systems (LHS) are emerging globally as a health policy approach to health system design and improvement [1, 2]. LHS can be defined broadly as component parts (e.g., individuals, organizations, innovations) that interact to promote, restore, and maintain health, while making connections between past actions, their effectiveness, and future actions to support continuous incremental health system improvements [1, 3]. LHS have been called out as a promising means to improve population health and value-based care [4] and heralded as a keystone in the delivery of person-centred and equitable healthcare [5, 6] because of their potential to enable continuous improvements in health outcomes and health services delivery. Several components within LHS enable continuous health systems improvement, such as the ability to access and harness clinical data, utilize information technology to deliver insights from these data into the care environment, understand and apply evidence-based innovations to deliver care, and engage patients and communities in all parts of the care journey [4]. Among this list of components is the notion of a learning routine which is intended to harness information and improve ongoing adaptation and sustainability of health innovations (stability and endurance of ingrained change) [7]. This learning routine is deeply entrenched into the social, scientific, technological, policy, legal, and ethical pillars of LHS [4, 8].

The definition of innovations within this context aligns with the World Health Organization’s definition, which includes new or improved solutions with the potential to accelerate positive health impact [9]—see Table 1 for a full list of terms and definitions in this article. In Friedman’s model of LHS, there are three core processes central to learning routines: (1) converting data to knowledge (D2K); (2) applying knowledge to influence system or innovation performance (K2P); and (3) generating new data through observation of system or innovation performance changes (P2D) [4, 8, 10]. Central to the operation of these processes is the concept of evaluation. Evaluation includes multiple indicators that measure different dimensions of LHS performance [4], including all efforts to assess the merit, impact, enactment, and experience of those interacting with health innovations. Ultimately, evaluation that is embedded into the everyday workflows of individuals and teams operating within LHS acts as an enabler to connect the processes of the learning routine [8, 10]. Evaluation is employed in D2K as a tool to identify problems, collect data, and transform data into contextualized information, ready to be interpreted and applied to the improvement process. In K2P, knowledge generated through evaluation is utilized to select areas for improvement, identify change indicators to be monitored, and specify appropriate actions to improve outcomes, and in P2D, the data harvesting and learning cycle begins anew. The essential function of evaluation within LHS is that it catalyzes the ability of health systems to learn from improvement efforts, supporting a trajectory of improvement [1, 11].

**Table 1:** Terms and definitions

Term	Definition
Learning health systems	A set of components (including people and innovations) which interact to promote, restore, and maintain health, while making connections between past actions, their effectiveness, and future actions to support continuous incremental improvements within the health system
Sustainability	The stability and endurance of engrained change of an innovation within a health system
Health innovations	New or improved solutions with the potential to accelerate positive health impact
Learning routines	Established cycles which transform data generated from healthcare practice-based activities to insights that are then re-applied to the clinical environment
Evaluation	Efforts to assess multiple aspects of LHS performance [4], including the merit, impact, enactment, and experience of those interacting with health innovations. Evaluations can be conducted at any stage of the innovation lifecycle and use a wide variety of methods to generate insight
Context	The backdrop of conditions in which interventions are implemented [12]. These conditions can be any circumstance which triggers and/or modifies a mechanism, including examples such as historical events, cultural norms, existing social networks, funding sources, participant characteristics, and opportunities or constraints offered by interventions [12]
Mechanism	Causal forces, including underlying entities, processes, or structures which operate in particular contexts to generate outcomes [12]
Outcome	The results of mechanisms operating in particular contexts

Learning is an important outcome in itself, but the ability to continuously learn from data-driven healthcare processes also supports the broader capacity of healthcare systems to sustain these innovations over the long term by continuously improving them, as well as to scale and spread successful innovations to different contexts [11]. The learning cycle of data harvesting, analysis, interpretation, and application supported through evaluation activities increases the likelihood of innovations transitioning into sustained practice by drawing continuous attention to the innovation and striving to improve the fit of the innovation within the dynamic context in which it is situated [11, 13].

Despite the central role that evaluation plays in learning and innovation sustainability within LHS, not all evaluations are equal in their ability to generate learning and improve innovation sustainability. In a recent systematic review, authors found 23 examples of LHS from across the globe using data to drive healthcare improvement, with benefits identified for patients, clinician-patient encounters, and health organizations and systems [10]. For example, the LHS model at The Ottawa Hospital redesigned twelve major processes in care for lung cancer patients to address delays from referral to treatment for new lung cancer patients [10, 14]. Data-driven learning cycles, enabled by evaluation in this project, included displaying locally generated performance data and provincial targets on dashboards to enable visibility of data trends and to spur appropriate corrective action [14]. Engaging staff within routinized learning cycles enabled The Ottawa Hospital to meet or exceed provincial targets in time to diagnosis and time to treatment, with results now having been sustained over several years [14]. In another example from a wound care initiative LHS among 12 facilities in the United States, learning cycles were operationalized by harnessing data from a clinical wound care data registry, which supported individual facility benchmarking with a national registry [15]. A purpose-built electronic health record and standardization of potential sources of bias across centres enabled clinical effectiveness research to be carried out within the LHS, with performance reports revealing learning opportunities within individual organizations [15].

Despite these examples of success, Greenhalgh and Russell argue that several factors place many health innovation evaluations at risk for failure [16]. For example, evaluations couched only in a positivist paradigm neglect rich contextual factors that can influence attainment and sustainability of innovation outcomes [16, 17]. Additionally, Greenhalgh and Russell postulate that other evaluation factors traditionally aligning with the positivist paradigm, such as evaluator objectivity and distance from the study phenomenon/innovation, do not actually constitute “good research” as traditionally thought [16]. Objectivity may in fact blind evaluators to the multitude of interacting and interdependent relationships that are key to understanding why some innovations succeed while others fail [16]. LHS are complex social systems in which the concepts of dynamism and adaptation of the system to an ever-changing context are embodied [1]. Thus, investing effort in the search for standardized evaluation mechanisms that produce predefined effects through “rational” behaviors may be utopian, if not futile, and attention should rather be focused on how evaluation enables the development of dynamic capacities for the continuous adaptation and improvement of health systems through knowledge flows [18].

When studying health innovations, the innovation itself as well as the dynamic and situated nature of the innovation may influence realized outcomes [12, 19]. To use an example from Wong and colleagues, a health promotion campaign promoting exercise as a means of preventing the onset of chronic disease could both (a) improve the exercise habits of a subgroup of the target audience, thus minimizing their risk of preventable disease, *and* (b) increase the anxiety levels of the ‘worried well’, thus increasing health service usage for this group and depleting healthcare resources for those who need them [12]. Evaluations of health innovations need to consider both intended as well as unintended outcomes, especially in complex health systems with multiple contextual factors to consider.

While the literature has begun to uncover underlying factors to health innovation evaluation success and failure within LHS, there is little consensus as to why certain evaluations seem to support learning and sustainability, while others impede it. It is our hypothesis that certain attributes of evaluation and the particular ways in which evaluations are enacted may influence the ability of evaluation to support or hinder learning and sustainability. Thus, we need to better understand the contextual factors that best support the drivers of health systems learning and sustainable innovation.

## What will this review add?

This realist synthesis will illuminate the ways in which evaluations of health innovations either support or detract from learning and innovation sustainability so as to inform the operationalization of learning- and sustainability-focused evaluations within LHS settings.

## Methods

### Approach

A realist synthesis (or realist review) is a complexity-compatible method of capturing, distilling, and drawing out information to answer questions of “what works, for whom, under what circumstances, and why?” [19]. Realist syntheses attend to the interplay between context, mechanisms, and outcomes by drawing out interdependencies between interventions, the people or organizations implementing them, and factors such as place, time, and social and political structures [20]. In realist terms, changes in context (C), activate different mechanisms (M), and thus produce different outcomes (O). The purpose of a realist synthesis is to use secondary data from documents and subject matter experts to develop testable theory(ies) about a program or intervention—often called a Program Theory (PT). A realist PT is accompanied by evidence-informed CMO configurations (CMOCs) or hypotheses. These CMOCs assist in theorizing why certain contextual factors are important, and how and why people respond to interventions (the mechanisms of change) [21]. In formulating and testing these hypotheses over the course of a realist review, the researcher seeks to explore causality beyond the narrow definition of the experimental paradigm of deterministic inputs and outputs [22].

In this review, the evaluation of health innovations is the ‘intervention’ under study. We believe that studying different evaluation approaches will help us to answer questions about what types of evaluations work to promote LHS learning and health innovation sustainability, for whom do they work, under which circumstances, and why? Given the complexity under which evaluations of health innovations in LHS are implemented, an approach that accounts for, embraces, and teases out complexity such as a realist synthesis, is an appropriate method. This realist protocol is being reported in accordance with the Preferred Reporting Items for Systematic Review and Meta-Analysis Protocols (PRISMA-P) Statement (Additional file 1).

### Procedures

Our review will follow methods described by Pawson and colleagues [23] and Emmel et al. [24] to move through five stages of (1) scoping the review, (2) building theories, (3) identifying the evidence, (4) evidence selection and appraisal, and (5) data extraction and synthesis. Though these steps are listed sequentially, the steps may at times overlap or proceed in parallel, according to the needs of the project. To date, we have completed the scoping and theory building stages, and have begun to identify evidence to develop CMOCs that correspond with our PT.

### Step 1: scoping the review

According to Pawson et al., Scoping the Review begins with identifying the research question, specifying the nature of the intervention being studied, and articulating key theories related to the intervention to be explored by drawing up a ‘long list’ of theories through exploratory searching [23]. To begin, our group developed two project teams—an Expert Committee (CSG, ÉCB, JS, LJ, MBird, MM, WPW) comprised of interdisciplinary subject matter experts in fields related to the scope of this review, such as learning health systems, evaluation, health innovation, sustainability, as well as realist methodology; and a Task Team (FB, MBhalla, TJ) led by MBird, comprised of health services research doctoral students and research assistants. The Expert Committee’s role is to provide ongoing conceptual and methodological guidance and feedback throughout the review, and the Task Team conducts the review work such as screening, appraising, coding, and extracting the evidence.

**Fig. 1:**
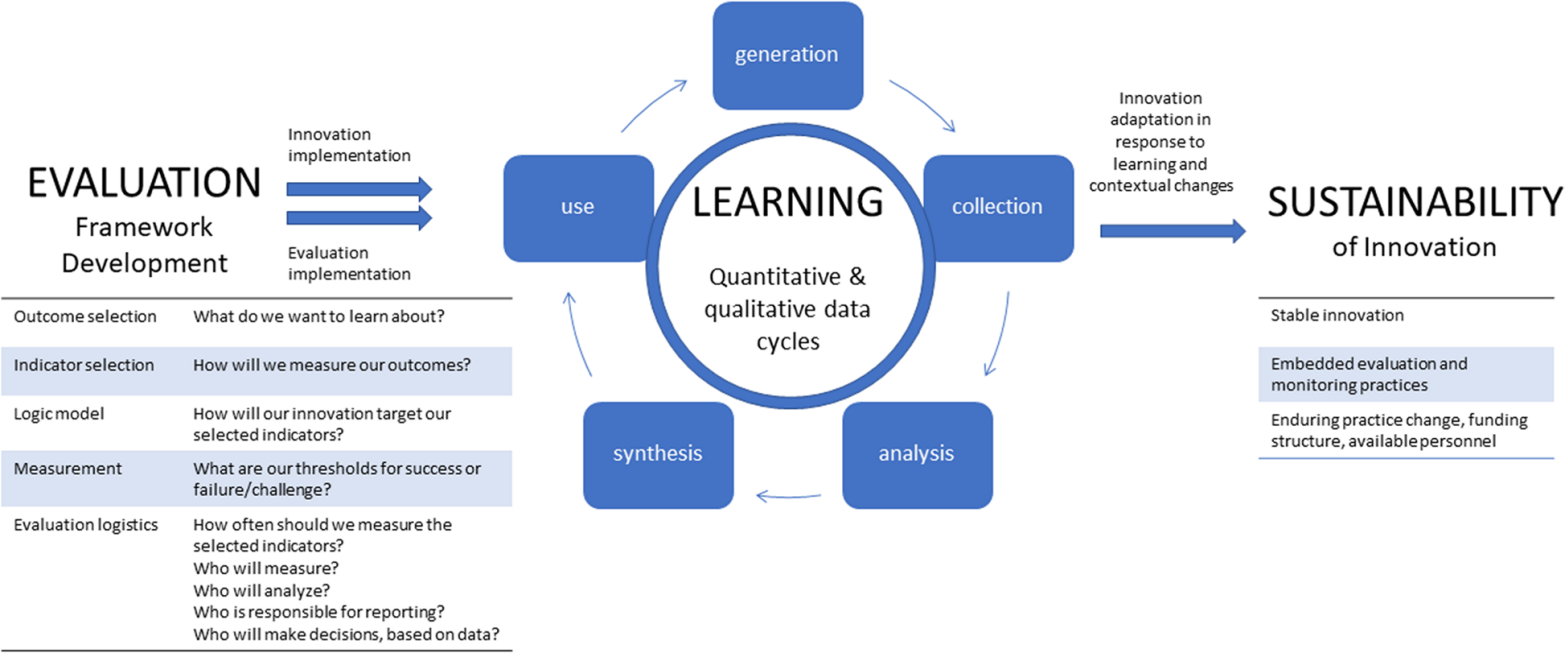
Initial conceptualization of the evaluation-learning-sustainability link

Together, the Expert Committee and Task Team first sought to clarify the scope of this synthesis. Twenty-five foundational articles from the fields of LHS, evaluation, and health innovation sustainability were identified from the personal libraries of the authors of this paper, deliberation and discussion among authors, as well as scoping searches of the literature that revealed highly-cited relevant articles to this topic. The theses or propositions contained within these articles were extracted into charts which showed areas of conceptual overlap in evaluation, learning, and sustainability. Next, the lead author created a diagram representing the theoretical and conceptual linkages between evaluation, learning, and sustainability (Fig. 1). Based on a discussion of Fig. 1 with the Expert Committee, areas were identified for further exploration of conceptual linkages between evaluation, learning, and sustainability.

The agreed upon research question was: what are the contextual factors associated with different evaluative approaches that trigger underlying mechanisms associated with LHS innovation and sustainability? In realist terms, ‘what works for whom, why, and under what circumstances’?

### Step 2: building theories

For the theory-building stage [25], the lead author (MBird), used the 25 foundational articles and additional literature obtained through scoping searches to extract and synthesize findings into a series of 14 concept clusters that formed the basis of our Initial Program Theory (IPT). In line with realist methodology, these clusters were then reviewed during two meetings with the Expert Committee in which IPTs were introduced to the group, refinements were suggested, and new IPTs were generated by considering group tacit knowledge, and using creative brainstorming exercises and retroductive thinking—combining induction, deduction, and insight to identify causal mechanisms behind patterns [25]. We now have a co-developed IPT, an initial broad sampling frame, and potential search strategies. The list of 14 concept clusters and our IPT can be found in Additional file 2.

### Step 3: identifying the evidence

The next stage of this realist synthesis will identify additional relevant evidence against which we will develop our IPTs. Searching in realist syntheses is intricate, iterative, and closely linked with other stages [23]. Searching involves conducting initial background searches to get a ‘feel’ for the literature, building to progressively focused searches aimed at teasing out contexts and mechanisms relevant to theories being explored [23]. We are currently partway through our evidence identification process, which is being conducted iteratively and purposively, with initial broad searches informing the need for and conduct of more specific and refined searches. By progressively extending and refining our search strategies, we will iteratively assess the extent to which our research question has been sufficiently answered.

#### Primary search

To begin our primary search, we searched the MEDLINE (National Library of Medicine) and Embase (Elsevier) academic bibliographic databases, using a combination of MeSH terms and key words conceptually centred around ‘evaluation’, ‘learning’, and ‘sustainability’, and ‘healthcare’. We included any type of article, book, dissertation, or report describing any form of evaluation (research, quality improvement, process evaluation, retrospective review) of healthcare innovations. We used the ‘.tw,kf.’ controlled vocabulary in both MEDLINE and Embase to capture evidence that contained our concepts of interest in the title, abstract, or key words to increase relevance. We limited the date of the search to ‘2013-present’ to account for the explosive growth of interest in the sustainability of health innovations over the last decade. A sample MEDLINE search can be found in Additional file 3.

#### Complementary searches

In keeping with established realist methods, our search includes opportunistic forays into the literature to capture as many relevant studies as realistically possible. These complementary searches are to be implemented iteratively as part of the evidence selection and appraisal process [24]. Specifically, we will implement: hand-searching of highly relevant journals (e.g., *Learning Health Systems, Implementation Science, Evaluation & the Health Professions, BMC Health Services Research*), scoping the grey literature (for example, websites for *Agency for Healthcare Research and Quality* [https://www.ahrq.gov/]*, The Learning Healthcare Project* [https://learninghealthcareproject.org/]*, Nuffield Trust* [https://www.nuffieldtrust.org.uk/]*, Alliance for Healthier Communities* [https://www.allianceon.org/]), contacting authors of relevant conference publications when a full-text manuscript cannot be found, forward and backward citation tracking, and searching for linked manuscripts of relevant evidence. These ‘snowball sampling’ techniques, in which a smaller number of key references gradually build to a larger set of references, are methods known to be effective for capturing a wide breadth of relevant information in realist methodology [24, 26]. All citations will be exported to Covidence Systematic Review Software [27] and duplicates removed.

### Step 4: evidence selection and appraisal

Unlike a traditional systematic review, inclusion and exclusion criteria in a realist synthesis are based on the relevance of citations to IPTs, and not the interventions themselves [21]. Studies are appraised for their relevance, richness, and rigour, as well as their ability to contribute to our understanding of generative causation in the overall PT [28]. Therefore, even methodologically weak studies that are relevant to the IPTs may contain ‘nuggets’ of truth that are useful in developing, iterating, and adjusting the overall PT [29]. For example, methodologically weaker studies may contain relevant author insights that would not be captured if study type were an a priori screening criterion for excluding studies [29]. These nuggets are often not evident in article titles or abstracts but rather require full-text review to uncover them. Because of this, our Task Team will complete a three-stage screening process—first, title and abstract screening will be completed to yield articles that are health innovation evaluations or manuscripts focusing on some aspect of health innovation evaluation, sustainability, or learning health systems. Articles will be limited to English language for feasibility. Articles will next be assessed at the full text level using specific keyword search strings and synonym lists to assess for discussions of learning and/or sustainability. Articles will be excluded if they are abstracts only with no discoverable full text article. Many articles that were included at the title and abstract level are expected to be reports of evaluations of health innovations that do not explicate a link to learning and/or sustainability and will be excluded at this stage. Finally, a second round of full text screening will be completed by two independent reviewers to assess for relevance of manuscripts to the conceptualization of how evaluation influences learning and/or sustainability. Articles at this stage will be assessed for relevance to the overall research question, with the aim of including evidence that can empirically test, refine, or revise the IPTs. Manuscripts that pass this stage of review will be imported into Dedoose qualitative software application for data extraction [30].

### Step 5: data extraction and synthesis

#### Coding and data extraction

We will use methods suggested by Dalkin and colleagues for using computer assisted qualitative data analysis software to refine and test IPTs to guide our data extraction and synthesis [31]. First, we will create a code tree in Dedoose qualitative analysis software using an abbreviated version of the 14 clusters with a linked memo to fully explicate the code. These clusters may represent potential context-mechanism-outcome configurations (CMOCs) pertaining to evaluation, learning and sustainability. Next, manuscripts that pass the second round of full-text screening will be imported into Dedoose and coded using these concept clusters as a deductive analytic framework. We will also create an ‘Other’ code that will allow us to capture important information outside of the concept clusters. Coded text from articles will be excerpted and filed under the corresponding section of the code tree in Dedoose, allowing for each code to be viewed and analyzed in isolation with its corresponding excerpts.

Excerpts filed under each code will be exported to Microsoft Excel for analysis [32]. An analytic Excel sheet will be created using columns for each concept cluster to develop CMOCs and to identify CMOC exemplars from the text (i.e., for rigor, relevance and richness [23]). Constant comparative analysis [33] will be used to examine the evolving PT and CMOCs for agreement or divergence among the coders. All data extractors are experienced in qualitative data analysis and coding techniques (MBird, TA, FB).

#### Sense-making and consensus

A synchronous interim analysis meeting will be held with the Expert Committee after the first 30 articles have been coded to discuss the evolving PT and CMOCs. In this meeting, the Expert Committee will work together to review the coding progress and map the CMOCs to the diagram (see Fig. 1) to ensure the evaluation-learning-sustainability links across the CMOCs (see Fig. 1). A goal will be to create a parsimonious number of unique CMOCs from the original 14 conceptual clusters. The Expert Committee will assist in identifying areas for further clarification, which will direct the final focused searches of the literature to refine and revise the final PT and CMOCs [23]. The searching, coding, and consensus discussions between coders and the Expert committee will continue iteratively until a final consensus is reached with respect to the refined PT and CMOCs and supportive text (i.e., CMOC exemplars).

## Discussion

This study will use a realist synthesis approach to explicate ‘what works, for whom, why, and under what circumstances’ in terms of the influence of evaluative characteristics and approaches on learning and innovation sustainability outcomes within LHS settings. The use of a realist review will allow for exploration and theorizing about the contextual factors and underlying mechanisms that make evaluations ‘work’ or not work to support learning and sustainability. The final PT and CMOCs with accompanying narrative will constitute the substantive content of the final report. Completion of the realist synthesis will explain how evaluative approaches support or detract from learning and innovation sustainability in healthcare. If practical, the final CMOCs will be translated into a series of recommendations for evaluators interested in supporting learning and sustainability within LHS settings. In the final synthesis, our manuscript will be reported using the Realist And Meta-narrative Evidence Syntheses: Evolving Standards (RAMESES) quality and publication standards [22]. Depending on results, we will attempt to organize findings into a set of recommendations for evaluations that support learning and innovation sustainability within LHS settings.

Our results and recommendations will have implications for both academic and practice spheres within LHS. Because evaluations of health innovations may take many forms and be implemented longitudinally throughout the innovation lifecycle, our results have implications for the phases of innovation design, implementation, and sustainability. From an academic perspective, the recommendations for optimizing evaluation for learning and sustainability in each of the aforementioned innovation phases could be further studied. There is also opportunity for the PT and CMOCs to be evaluated through field applications to test their effectiveness in promoting learning and sustainability of health innovations.

### Supplementary Information

**Additional file 1.** PRISMA-P 2015 Checklist.

**Additional file 2.** Concept Clusters & Initial Program Theory (IPT).

**Additional file 3.** Sample MEDLINE Search.

## Data Availability

The datasets used and/or analyzed during the current study are available from the corresponding author on reasonable request.

## Abbreviations

LHS: Learning Health Systems
D2K: Data to knowledge
K2P: Knowledge to performance
P2D: Performance to data
CMO: Context, mechanism, outcome
PRISMA-P: Preferred Reporting Items for Systematic Review and Meta-Analysis Protocols Statement
IPT: Initial Program Theory
PT: Program Theory
MEDLINE: Medical Literature Analysis and Retrieval System Online
RAMESES: Realist and Meta-narrative Evidence Syntheses: Evolving Standards
