# Supplementary material for: Exploring the impact of evaluation on learning and health innovation sustainability: protocol for a realist synthesis
Source: Syst Rev. 2023 Oct 6;12:188. doi: 10.1186/s13643-023-02348-5 (PMC10557319; doi:10.1186/s13643-023-02348-5)
Supplement: Supplementary file 2 — Additional file 2. Concept Clusters & Initial Program Theory (IPT). [file 13643_2023_2348_MOESM2_ESM.docx]

**Additional File 2. Concept Clusters & Initial Program Theory (IPT)**

|  | **Title** | **Concept Cluster** |
| --- | --- | --- |
| ***Topic: context of evaluation*** | | |
| 1 | Punishment vs. learning | Evaluation is not neutral. The purpose of evaluative activities is to identify and scope “good” versus “poor” performance, for some overarching purpose. Depending on the interpretation of that purpose by those involved in implementing interventions, different reactions may be triggered. If the purpose of evaluation is perceived to be punitive or surveillant in nature, these individuals may be more likely to avoid and/or resist evaluation exercises, because they may fear that their individual or team performance will be scrutinized or punished. If, however, evaluative efforts are perceived by staff to be directed toward organizational learning with a mindset of innovation improvement, then staff may be more likely to embrace or engage in evaluation exercises, because they believe that they have a role in achieving these positive outcomes and do not fear punitive measures being taken as a result of evaluations. |
| 2 | Risk | If organizations are highly-risk averse, then they may be less willing to capture some forms of unpredictable data - such as emergent data (as opposed to indicators specified *a priori*) - as part of evaluations because they have less control over what happens after those data are collected. The purpose of evaluation for risk-averse organizations may lean toward the identification and control of poor performance or poorly-performing individuals or teams. If organizations are more risk-tolerant, they may be open to collection, interpretation, and use of these unpredictable data. For risk-tolerant organizations, the purpose of evaluation may lean toward learning and value-creation. |
| 3 | Co-production | If innovations and evaluations are co-produced in an open context, with greater inclusive and transparency, then the perceived value and feasibility of the innovation is increased because people affected by the innovation were part of understanding the problem, as well as designing and testing solutions. Co-production also increases the trust that individuals have in the accuracy and meaningfulness of the data. However, co-produced innovations can also create challenges with regard to reconciling competing views and interests and establishing clear lines of accountability and control towards evaluation. |
| 4 | Evaluation education | If individuals are given ongoing and audience-specific education and training about evaluation, they are more likely to stay engaged in evaluation activities because they recognize the organizational investment in them and their learning. This investment in evaluation education for individuals signals that evaluation is an organizational priority, increasing the likelihood of individual participation. |
| 5 | Individual and organizational learning | As an outcome of evaluation, learning at an individual level influences organizational learning because individuals contribute their knowledge gained from various sources to a shared learning ecosystem. The life experiences of the individual, their exposure to and practice with learning from various sources, and their willingness and ability to contribute to the shared learning ecosystem will influence the learning that occurs as a result of evaluation at an organizational level. To this end, organizational structures must be in place to enable the development of a productive learning ecosystem for individuals, including organizational processes and procedures for knowledge contribution by individuals, and repositories for that information to be stored and used. |
| ***Topic: evaluation framework development and planning*** | | |
| 6 | Value of evaluation | Evaluation efforts must be perceived as valuable to maintain engagement. The benefits of efforts expended by staff to conduct evaluation work and produce evaluation deliverables must be clearly and continuously articulated in feedback loops. If staff can draw connections between their efforts directed toward evaluation and positive outcomes, such as improved system performance, patient care, team experience, individual success within an organization, or workflow efficiencies, their engagement with evaluation is likely to be sustained because they can make a direct connection between their participation and positive outcomes. If, however, evaluative efforts by staff are not perceived by staff to be valuable, for example, data collected or analyzed are not fed back to staff, or there is no perceived change or negative change in system performance, patient care, team experience, individual success within an organization, or workflow efficiencies, then staff are likely to disengage with evaluation activities because they cannot make the connection between their efforts and positive outcomes. |
| 7 | Innovation lever | If evaluation serves as a lever to generate a productive evidence ecosystem to stimulate innovation, then evaluation is also a learning tool because it helps to shift the focus from practice and operational improvement to co-producing, sustaining, scaling, and spreading innovation. |
| 8 | Choosing the right evaluation method | Evaluation can only act as a mechanism for learning and innovation sustainability if the evaluation is designed to capture the right outcomes, in the right way, from the right sources, to enable learning to take place. Careful scrutiny of evaluation frameworks for their fit with learning outcomes and an openness to pragmatically designing evaluations to fit the desired learning outcomes is necessary to enable learning and sustainability outcomes. |
| ***Topic: learning cycles*** | | |
| 9 | Learning intention | For evaluation activities to produce learning, data generation, collection, analysis, reporting, and use must be implemented purposefully with the intention of learning. If teams habitually engage in these data cycles with the end point of utilizing knowledge gained to take action (including maintaining, iterating, adjusting, or stopping an intervention, according to the data), evaluation can produce learning by supplying evidence on which to make decisions. However, if the data cycle is broken (e.g., data are collected but not analyzed or used to generate change where needed), evaluation will not lead to learning. |
| 10 | Timely feedback | If individuals regularly receive timely feedback about evaluation findings, they will continue to engage in the evaluation process (gathering, analyzing, utilizing data), because they appreciate the utility of data to improve practice. The timing of evaluation feedback must be based on what is useful to the team doing the work of implementation and evaluation and should be flexibly adapted if needed. |
| 11 | Learning actualized in “use” | Learning is actualized in the “use” of knowledge gained being applied to practice, which serves to further inform individual and organizational learning, as well as optimize the innovation for sustainability. |
| 12 | Visibility of learning | Evaluation modifies the way that we learn by making visible the processes of learning, which allows for scrutiny as to whether necessary components for learning are missing. |
| ***Topic: innovation and evaluation sustainability*** | | |
| 13 | Continuity of evaluation | For evaluation to lead to sustainability, evaluation and learning activities must become engrained in workflows by adopting and adapting practice and policies that allow evaluation to become routinized. Continuous evaluation and learning allow for continual intervention adjustments to be made to adapt to changing context and maintain the intervention fit within dynamic circumstances over the long term. Conversely, short term evaluations or learning activities will not necessarily contribute to intervention sustainability. |
| 14 | Resources for learning and sustainability | If people have an opportunity (time, resources) to review and reflect with their results/data then they will be more likely to be able to learn and sustain the entire evaluation/learning process. Regular learning review activities that are routinized in individual, team, unit, or system workflows enhance the sustainability of the evaluation and learning process. |

**IPT:** Evaluation helps to sustain an LHS when it contributes to establishing an enabling environment, with necessary and supportive resources that help drive an ongoing process of learning. Learning within an LHS helps to sustainably achieve outcomes while continuously building the enabling learning environment.
