# Supplementary material for: Exploring the impact of evaluation on learning and health innovation sustainability: protocol for a realist synthesis
Source: Syst Rev. 2023 Oct 6;12:188. doi: 10.1186/s13643-023-02348-5 (PMC10557319; doi:10.1186/s13643-023-02348-5)
Supplement: Supplementary file 3 — Additional file 3. Sample MEDLINE Search. [file 13643_2023_2348_MOESM3_ESM.docx]

**Additional File 3. Sample MEDLINE Search**

**Database: OVID Medline Epub Ahead of Print, In-Process & Other Non-Indexed Citations, Ovid MEDLINE(R) Daily and Ovid MEDLINE(R) 1946 to Present**

***November 16, 2022***

| **Search Number** | **Reference Concept: Evaluation** | **Hits** | **Totals** |
| --- | --- | --- | --- |
| 1 | Evaluation Study/ or Program Evaluation/ | 320426 |  |
| 2 | Qualitative Research/ | 77647 |  |
| 3 | Research Design/ or Nursing Research/ | 135335 |  |
| 4 | Evaluat*.tw,kf. | 4208172 |  |
| 5 | ((formative or summative or process or heuristic or developmental or framework) adj2 evaluat*).tw,kf. | 22816 |  |
| 6 | (pilot or feasibility or questionnaire or survey or interview or usability or realist or pretest or pre-test or posttest or post-test or crossover or cohort or cross-sectional or crossover or trial or (case adj2 study) or (action adj2 research) or (focus ajd1 group) or quantitative or qualitative or (mixed adj1 method*) or usability or (use* adj2 test*) or RCT ).tw,kf. | 4199986 |  |
| 7 | ((randomi$ed or control* or stepped or wedge or clinical or pragmatic or cluster) adj2 trial).tw,kf. | 197949 |  |
| 8 | 1 or 2 or 3 or 4 or 5 or 6 or 7 | | 7531401 |
| **Search Number** | **Reference Concept: Learning** | **Hits** | **Totals** |
| 9 | Learning/ or Quality Improvement/ or Organizational Culture/ or Organizational Innovation | 148722 |  |
| 10 | ((Organi$ational or social) adj2 learning).tw,kf. | 5366 |  |
| 11 | (Learn* adj2 (health* system)).tw,kf. | 797 |  |
| 12 | ((Double or triple) adj2 loop learning).tw,kf. | 46 |  |
| 13 | ((Meta or high) adj2 learning).tw,kf. | 1450 |  |
| 14 | 9 or 10 or 11 or 12 or 13 | | 154816 |
| **Search Number** | **Reference Concept: Sustainability** | **Hits** | **Totals** |
| 15 | (Sustainability or sustainment or scale or scale-up or spread or expand or expansion or dissemination or diffusion or extension).tw,kf. | 1863126 |  |
| 15 | 15 | | 1863126 |
| **Search Number** | **Reference Concept: Healthcare** | **Hits** | **Totals** |
| 16 | "Delivery of Health Care"/og [Organization & Administration] | 22507 |  |
| 17 | Health or healthcare or (health ajd1 system) or clinic or (health adj2 service) or hospital or (primary adj1 care) or (emergency adj1 department) or (emergency adj1 room) or (doctor* adj1 office) or unit | 4057805 |  |
| 18 | 16 or 17 | | 4064054 |
| **19** | **8 and 14 and 15 and 18** | | **3449** |
| **20** | **Lim: 2013-Current** | | **2688** |
